# Enzyme function and evolution through the lens of bioinformatics

**DOI:** 10.1042/BCJ20220405

**Published:** 2023-11-22

**Authors:** Antonio J. M. Ribeiro, Ioannis G. Riziotis, Neera Borkakoti, Janet M. Thornton

**Affiliations:** European Bioinformatics Institute, Wellcome Trust Genome Campus, Hinxton, Cambridge CB10 1SD, U.K.

**Keywords:** biological databases, catalytic sites, enzyme evolution, enzyme mechanism, ligand binding, protein structure

## Abstract

Enzymes have been shaped by evolution over billions of years to catalyse the chemical reactions that support life on earth. Dispersed in the literature, or organised in online databases, knowledge about enzymes can be structured in distinct dimensions, either related to their quality as biological macromolecules, such as their sequence and structure, or related to their chemical functions, such as the catalytic site, kinetics, mechanism, and overall reaction. The evolution of enzymes can only be understood when each of these dimensions is considered. In addition, many of the properties of enzymes only make sense in the light of evolution. We start this review by outlining the main paradigms of enzyme evolution, including gene duplication and divergence, convergent evolution, and evolution by recombination of domains. In the second part, we overview the current collective knowledge about enzymes, as organised by different types of data and collected in several databases. We also highlight some increasingly powerful computational tools that can be used to close gaps in understanding, in particular for types of data that require laborious experimental protocols. We believe that recent advances in protein structure prediction will be a powerful catalyst for the prediction of binding, mechanism, and ultimately, chemical reactions. A comprehensive mapping of enzyme function and evolution may be attainable in the near future.

## Introduction

**Figure 1.**
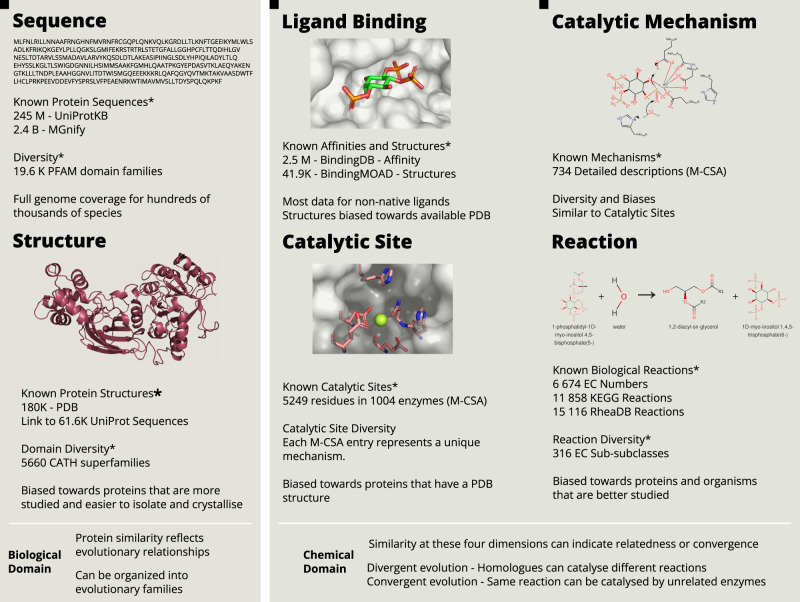
Six dimensions of enzymes, related databases and data diversity and biases. *The list of databases and resources is not exhaustive, but it aims to be representative of the type and amount of data available.

Lying at the interface between biology and chemistry, enzymes are complex subjects to study. To understand how they function, it is necessary to integrate diverse kinds of data, including amino-acid sequence [1], three-dimensional structure [2], knowledge about their catalytic residues [3] and co-factors [4], and the chemical reactions they catalyse [5,6]. Lastly, enzyme mechanisms [7], which are the sequence of bond changes and atom movements that happen in the active site during catalysis, present the fundamental explanation for how enzymes operate. Figure 1 provides an outline of the collective understanding of enzymes across these six dimensions and shows some of the databases containing this knowledge.

In addition to this complexity, enzymes should be viewed as changing entities. They have been evolving for billions of years, as shaped by natural selection, and across millions of species. The study of evolution and the aforementioned dimensions cannot be detached from one another. While genetic mutations govern and constrain changes in the enzyme sequence (the first dimension in Figure 1), natural selection acts primarily at the level of the biological function (the chemical reaction for enzymes, the last dimension in Figure 1). Additionally, like the challenge of mapping genomes to phenomes [8], establishing a causal link between sequence changes and catalytic activity in enzymes requires examining the intermediate dimensions.

An ideal knowledge base of enzyme function and evolution would consist of multiple maps, each representing one of the dimensions mentioned in Figure 1, and it would explain how these different aspects of enzymes relate to each other and how they have changed through time. It would provide explanations, or predictions, for how changing one dimension would alter the others. This would allow us to understand the historical and natural process of change, enzyme evolution, and would be a significant contribution for enzyme engineering [9,10] and drug development [11,12].

How far are we from being able to build such a comprehensive resource? UniProt [1], the most extensively annotated dataset of protein sequence data, currently covers the complete genomes of hundreds of thousands of species, a substantial and partially representative collection of all life on earth. At the same time, data coverage on other aspects of proteins, and in particular enzymes, such as structure, ligand binding, mechanism, or the reaction, is not as extensive. Nevertheless, equipped with what is already known about enzyme function and evolution, and increasingly powerful and diverse methods, we believe it should be feasible to use sequence data as the seed to populate all the other dimensions, all the way through to the enzyme reaction. The recent advances in predicting structure from sequence [13] demonstrate that this seemingly utopian vision may be within reach.

## Enzyme evolution

Like other biological components, enzymes are best understood in the light of evolution. Similarities between enzymes reveal their evolutionary relationships, with more similar sequences having diverged more recently. Whereas all organisms on earth share a common origin, the Last Universal Common Ancestor (LUCA) [14], proteins and enzymes can be grouped into several evolutionary families, which have, for the most part, separate evolutionary histories. Within families, enzymes present a remarkable degree of conservation, emphasising the significant selective pressure to preserve catalytic function. Certain structural folds and active sites, for example, are so well conserved that they can be traced back to LUCA [15]. Although estimations about the number and type of proteins in LUCA are uncertain [16,17], it is likely that a majority of them were enzymes. A recent consensus analysis, identified 199 enzymes among 366 possible ancestral proteins [18].

Since then, new enzymes, like all proteins, have mostly evolved by gene duplication and divergence [19]. Other genetic events, such as the fusion and swapping of domains, are rarer but also important, since they allow for larger evolutionary jumps and more significant changes of function [20]. Finally, *de novo* evolution of proteins from non-coding DNA is also possible, and not limited to the ancient past, as previously thought [21]. *De novo* proteins, are typically similar to small random peptidic chains, except for their improved solubility [22], and their physiological functions have been characterised for only a handful of cases, so they will not be discussed further in this review.

### Enzyme evolution by gene duplication and divergence

Following the first observations of proteins sharing similar sequences, suggesting homology and common ancestry, Susumo Ohno proposed a neofunctionalization model based on gene duplication and divergence [23]. This model proposes that after a random duplication event, one of the gene copies can diverge without compromising organism fitness, and that these mutations may eventually result in the acquisition of a new function. The importance of duplication and divergence for neofunctionalisation has been reinforced since, but alternative models have been proposed [24] to address some limitations of Ohno's model. Most notably, these alternatives take into account that deleterious mutations typically accumulate faster than gain-of-function mutations [25], which often lead to complete loss of function and eventual deletion of the duplicated gene.

**Figure 2.**
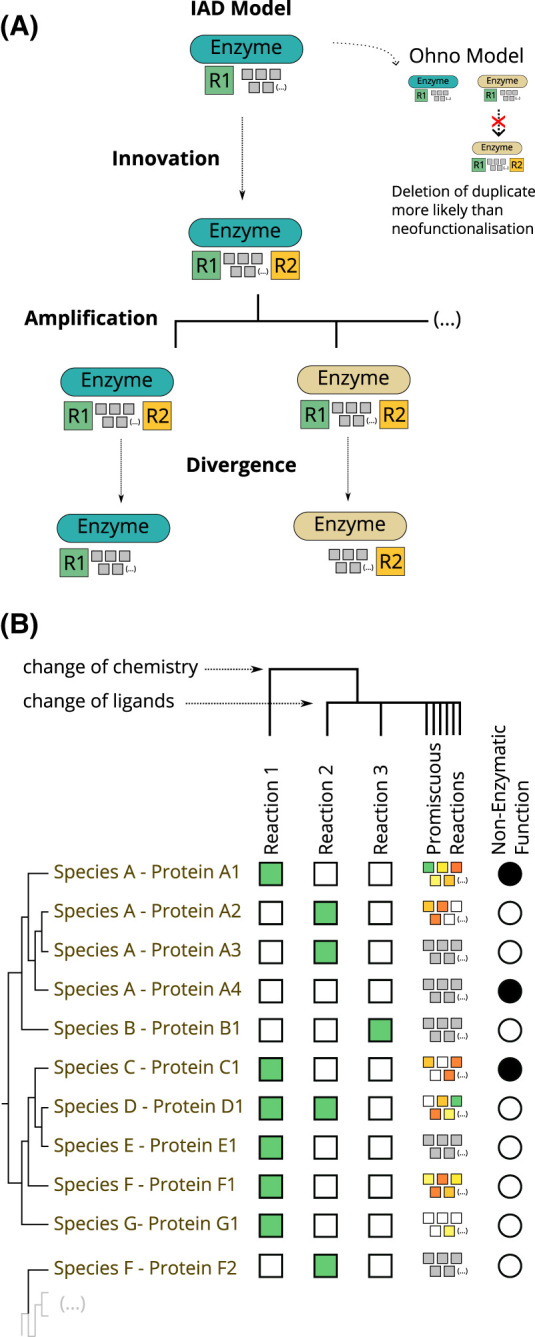
Models of divergent evolution and possible evolutionary relationships among extant enzymes. (**A**) The Innovation–Amplification–Divergence (IAD) model of enzyme evolution. Ohno's model is shown for comparison. A description of both models is given in the main text in the ‘Enzyme Evolution by Gene Duplication and Divergence' section. (**B**) Evolutionary and functional relationships between enzymes and non-enzymatic proteins. The colours of the squares are meant to indicate enzyme efficiency from green (high efficiency) to red (low efficiency). Grey squares indicate that the enzyme is likely promiscuous but was not tested for promiscuous reactions. Black circles indicate that the protein has a non-enzymatic reaction. The types of evolutionary relationships are discussed in the ‘Evolutionary and Functional Relationships Between Enzymes' section.

The IAD (Innovation–Amplification–Divergence) model, also called the Adaptive Radiation model [25,26], solves this apparent dilemma while also considering the significance of promiscuity for enzyme evolution. The sequence of events in the IAD model is depicted in Figure 2 and described below.

#### Innovation

During evolution, some mutations grant enzymes the ability to catalyse additional reactions that have no impact on fitness, since they are either too slow to affect metabolism or involve inaccessible substrates. These so-called promiscuous reactions are widespread [27] and they are considered a latent pool of innovation for evolution to use [27–29]. A promiscuous reaction might become important for fitness after a change in the organism's environment, such as the introduction of industrial chemicals in soils [30,31].

#### Amplification

The enzymatic efficiency for a new reaction is typically low and cannot be easily improved. Beneficial mutations for the new reaction often have negative effects for the old one (pleiotropy), resulting in an evolutionary impasse. Furthermore, distinct regulation of both reactions is impossible, unless they occur in different cells or tissues. The solution for these problems is the duplication or further amplification of the gene. In IAD, this amplification, defined as a selective expansion in the number of copies of a gene, is a favourable genetic event in itself, since it results in a larger number of enzymes and, ultimately, in an increase in the reaction turnover in the cell.

#### Divergence

After amplification, the copies of the gene are free to independently diverge. As some copies improve their catalytic efficiency towards one reaction, others are deleted from the genome as they no longer provide an advantage. Eventually, only two copies of the gene remain, each specialised in their chemical reaction and associated regulation.

### Expansion of enzyme evolutionary families

A family of related enzymes originated solely by duplication and divergence will have a unique common ancestor and can be organised in a well-ordered phylogenetic tree in a way that mimics the evolution of species and the tree of life. This hierarchy is complicated by domain recombination, so this is discussed separately below. While some enzyme families are very specific and tend to catalyse only one function across all the organisms where they are expressed, in other cases, the process of duplication and divergence leads to, over time, an increase in the number of members in protein families and also the number and variety of their functions. Some well-studied functionally diverse enzyme families include the haloacid dehalogenases [32], Glutathione Transferases [33], and the amidohydrolases [34].

Using structural classification systems like CATH [35] or SCOP [36] to identify distantly related homologues together with functional assignments, such as the enzyme commission (EC) [37], it is possible to categorise enzyme families in an encompassing scale. FunTree [38] is a resource that shows phylogenetic and functional relationships between enzymes based on CATH and EC, and has been used to study the general evolution patterns of enzymes. For example, among 379 enzyme families [39] for which the catalytic function can be assigned to a single structural domain, there are enzymes catalysing 2994 unique reactions, meaning that most types of reactions (at least according to the EC classification) have diverged from a common ancestor. The EC classification can also be used for a more detailed analysis. The EC hierarchy is composed by four levels: class, subclass, sub-subclass, and serial number. The three first levels are used to define the type of the reaction while the serial number specifies the substrates and products. Enzymes that have the same sub-subclass (the same first three EC numbers) and only differ in the last digit, catalyse essentially the same type of reaction on a different substrate. Most evolutionary changes observed in FunTree and similar datasets are at the fourth EC level. One can also use changes of EC class (the first number in the EC code) to find more radical changes of function, which for the mentioned 379 families account for <20% (18.6%) of the changes observed.

Illuminating as they may be, studies like these fall short of providing a casual explanation between the changes in the protein sequence and the observed functional changes (akin to limitations in genome-wide associations studies for the genome and phenome). To establish these causal links, a comprehensive analysis of the mutation's impact on the enzyme's structure, ligand binding, and catalytic mechanism is necessary. Evolutionary studies with this level of detail and across families are rare [40] because these analyses are difficult to automate, in particular when considering mechanistic data. However, as we discuss in the ‘Enzyme Mechanism' section, we have recently made some progress in systematising the knowledge about enzyme mechanisms into ‘rules of enzyme catalysis', which might be a future foundation for such studies.

### Enzyme evolution by recombination of domains

The duplication models discussed above assume that an entire gene is duplicated, followed by independent changes in each copy. However, some genetic events can also lead to the insertion of genetic material from some genes into or next to other genes. Proteins domains are regions of the protein that fold independently and usually have a well-defined function. These domains serve as evolutionary units because genomic events that do not copy or move the entire domain are likely to disrupt its folding and function, rendering the resulting protein inactive. Throughout evolution, domains with distinct functions have been combined in different ways to create fully functional proteins [41,42].

The recombination of domains is also an important source of innovation in enzyme evolution. New domains can alter substrate specificity, regulate binding or catalytic activity, change the catalytic function, or simply add independent catalytic activities, resulting in multifunctional enzymes [43]. Furthermore, many enzymes that use co-factors evolve by combining the co-factor binding domains with other domains that bind the substrate or provide additional catalytic machinery. Notable examples include the Radical S-adenosylmethionine (SAM) superfamily [44], and FAD binding enzymes, such as Flavin dependent nitroreductases [45] and monooxygenases [20] where, interestingly, the sequence of the co-factor binding domains (but not the structure) is found interlaced with the sequence of the substrate binding domain.

Comprehensive and automated studies on the evolution of enzymes by domain recombination are challenging because the annotation of function in protein databases is traditionally given to the whole sequence. Preferably, one would want to know which function is contributed by each domain. The PDBe Knowledge Base and associated data sources [46], which provide residue-level annotations, might be a good starting point for future studies.

### Convergent evolution

The same selective pressure keeping catalytic residues and mechanisms extremely well conserved during evolution, also leads to cases of convergent evolution, where enzymes evolve similar catalytic capabilities independently. The most clear-cut examples of convergent evolution in catalysis, are enzymes that have different structural folds but can catalyse the same overall reaction (sometimes called Non-homologous Isofunctional Enzymes) [47]. From a bioinformatics point of view, for annotated enzymes, these can be detected by searching for enzymes that have a different CATH code (or similar evolutionary classification), but the same EC number. In the FunTree study mentioned above [39], it was observed that 59% of EC reactions are catalysed by proteins belonging to at least two CATH superfamilies, suggesting that convergence of chemical function is surprisingly common.

**Figure 3.**
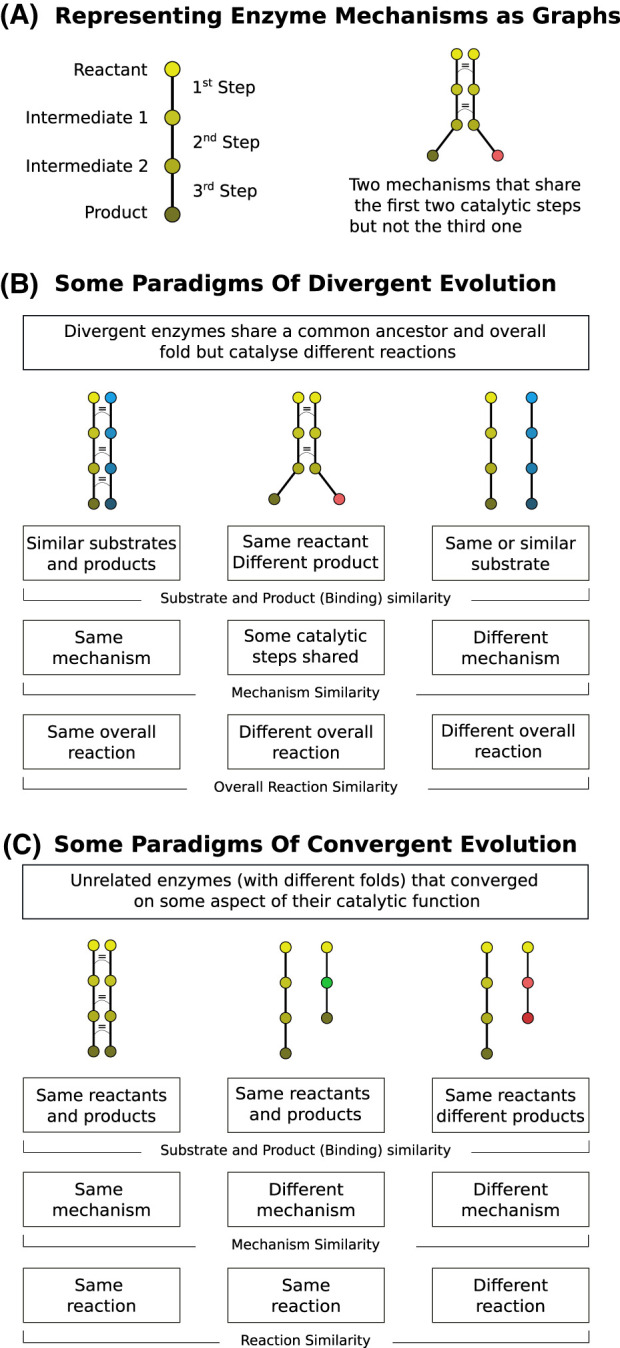
Some paradigms of enzyme evolution as defined by the similarities between the different dimensions of enzymes. (**A**) Mechanisms are represented as graphs where nodes (circles) represent stable configurations of the active site along the mechanism (reactants, intermediates and products) while edges (lines) represent the catalytic steps. Two mechanisms can be compared by showing their graph representation side by side. An equal sign is used to represent steps with a high degree of similarity. Nodes with the same colour represent the same ligand (substrate or product) in both mechanisms. Transformations along the mechanism are indicated with a change of gradation to darker colours or complete change of colour when the transformation between the two enzymes are different. (**B**) Three paradigms (among other possibilities) of the divergent evolution of enzymes. Enzymes might evolve to (from left to right): catalyse the same reaction using the same mechanism on a different substrate; catalyse a different reaction on the same substrate by a partial change in the mechanism; catalyse a completely unrelated reaction on the same or different substrate. (**C**) Three paradigms (among other possibilities) of the convergent evolution of enzymes. Enzymes with unrelated ancestry might converge to (from left to right): catalyse the same reaction using a similar mechanism; catalyse the same reaction using another mechanism; bind the same substrate to perform unrelated reactions.

Convergence can happen on different levels, as illustrated in the third panel of Figure 3. Complete convergence would be an example where the two enzymes catalyse exactly the same overall reaction (which implies having the same substrate and products) using an identical set of catalytic residues and reaction mechanism. Convergence only at the reaction level would mean that the enzymes catalyse the same reaction using a different mechanism and catalytic residues. It is also possible that enzymes catalyse a common catalytic step on different substrates or have converged to bind the same substrate or co-factor but catalyse a different reaction. Once more, a detailed account of the convergent evolution of enzymes would need to consider similarities at these different levels. This is even more important in the study of convergence than divergence because the lack of sequence similarity means that most of these relationships (such as mechanistic similarities) might be hidden in the data.

### Functional and evolutionary relationships between enzymes

The complex interplay between the evolution of enzymes and the chemistry they catalyse leads to a rich tapestry of possible sequence-function(s) associations. For example, one enzyme can catalyse multiple reactions, differing in their substrates or overall chemical transformations, and the same reaction can be catalysed by related or unrelated enzymes. The second panel of Figure 2 shows some of the different possibilities and Figure 3 shows how these can be explained in terms of the underlying binding capabilities, catalytic machinery, and the reaction mechanisms.

All enzymes catalyse at least one chemical reaction that is important for the fitness of the organism. This might be called the enzyme's primary or native reaction. Some enzymes (protein D1 in Figure 2, for example) are able to catalyse more than one reaction (or the same reaction on different substrates) where the additional reactions are also important for fitness. This can be a well specified secondary reaction, or the case of broad-specificity enzymes, which are able to catalyse the same type of reaction across a range of substrates, as in the case of detoxifying enzymes.

Promiscuous reactions, on the other hand, are reactions that enzymes are able to catalyse but that are currently irrelevant for biological function and the fitness of the organism (there are other definitions of enzyme promiscuity, but we think this is the most useful) [27]. For example. these might be reactions that are too slow to have a metabolic impact, or reactions that involve substrates that do not exist in the organisms or their environment. Although not immediately important for fitness, promiscuity is crucial for the evolvability of enzymes, as explained above [29,48]. It is increasingly clear from substrate profiling studies that most, if not all, enzymes are promiscuous.

Enzymes might also perform non-catalytic functions. When the non-catalytic functions are independent from the catalytic activity, these are sometimes called moonlighting enzymes [49]. Conversely, pseudoenzymes (protein A4 in Figure 2) are proteins that do not have any catalytic function but are evolutionarily related to enzymes [50]. Typically, pseudoenzymes evolve from a catalytic ancestor that has lost its catalytic function [51].

Orthologous enzymes (proteins A1 and C1 in Figure 2, for example) are homologous proteins that have diverged following a speciation event and keep catalysing the same primary reaction. Paralogous enzymes (A1 and A2) arise from gene duplication within the same genome and evolve to catalyse different functions. Isozymes (A2 and A3) are enzymes in the same organism that catalyse the same reaction but might have differential regulation and expression, which justify the presence of a duplicate. Convergent evolution is at play when unrelated enzymes catalyse the same reaction (A2 and F2). The correct identification of all these evolutionary relationships is crucial to predicting the function of uncharacterised enzymes [52].

### Evolution as an algorithm

As a search algorithm with the goal of finding catalytic proteins for a host of chemical reactions, enzyme evolution has several biological constrains, which limit the potential solutions it can find. Point mutations, the most common genetic event, restrict the size of potential changes to one residue position and to a small selection of the 20 amino acids (due to the genetic code, most amino acids changes are unreachable after mutating only one nucleotide). Series of mutations, which can be thought as a walk through the sequence space, cannot go through states where the activity of the enzymes is compromised or, depending on the selective pressure, even slightly lower. This means that the algorithm can get stuck in local maxima of fitness, and better maxima might be inaccessible because there is no favourable path to reach them (Evolution does not have foresight). One of the advantages of rational enzyme engineering is precisely the ability to make targeted jumps to parts of the sequence space that are unreachable to natural or even directed evolution.

The complexity of enzymes makes this a difficult search problem. Each of the dimensions discussed in this paper represent competing evolutionary goals, such as the maintenance of structural fold and stability or the enhancement of binding and the catalytic rate [45]. This results in an intricate evolutionary space where the effect of pleiotropic mutations and epistasis is significant [53]. It also explains why small changes and the overlap of catalytic functions (promiscuity) are so common, as opposed to large functional jumps, which will likely be deleterious. Finally, the presence of competing goals, coupled with the absence of a need for optimising beyond selective pressure, also justifies why evolution tends to produce enzymes that are ‘good enough’ rather than perfect.

Epistasis refers to the differential effect of mutations in one position being dependent on mutations in other positions of the same or different gene [54]. Epistasis is a reflection of the vast and multidimensional sequence/fitness space, where a particular mutation can only be called beneficial or deleterious (for enzyme activity, for example) in the context of a specific sequence, and where the role and importance of each position is not absolute but dependent on the environment. Pleiotropy refers to any genetic variants that affect more than one phenotype [53]. Pleiotropic mutations, in the context of enzyme evolution, are mutations that improve the ability of the enzyme to catalyse one reaction, while being detrimental to the other. These effects might be noticed at the level of the binding, the catalytic rate, or the overall mechanism. Pleiotropic mutations are an important reason for the necessity of the duplication of genes (together with independent regulation), because in most cases it is impossible to find a particular sequence that can effectively catalyse the two reactions of interest with optimal rates and expression.

## Mapping the dimensions of enzyme catalysis

**Figure 4.**
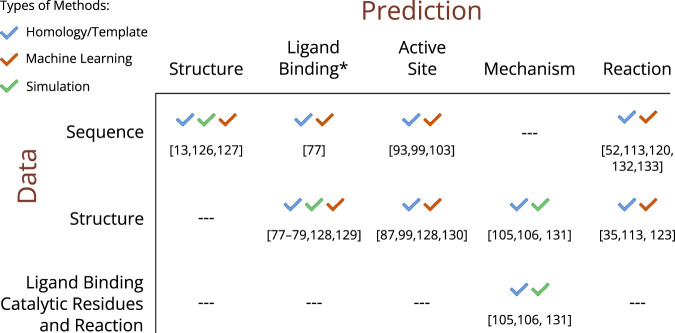
Overview of existing computational methods used to predict the dimensions of enzymes based on known data [13,35,52,77–79,87,93,99,103,105,106,113,120,123,126–133]. *Ligand Binding refers both to the identification of ligand binding sites and the prediction of native ligands and their binding poses.

Information about the various dimensions of enzyme catalysis can be found scattered throughout the literature but also consolidated in several databases, each dedicated to specific types of data [46]. In addition to providing a centralised location for accessing information, databases offer the added benefits of normalisation and structured data models, which facilitate analysis and the re-use of data. Prediction methods, typically trained or tested against these data, allow researchers to use certain data points (like sequence) to fill the gaps in knowledge about other data (such as structure). Figure 1, together with the sections below, provide an outline of the collective understanding of enzymes across the six dimensions and shows some of the databases containing this knowledge. We also discuss how some of the missing data can be predicted from computational methods (summarised in Figure 4) and give a non-exhaustive account of some of these tools for each dimension.

### Sequence

Among the 6 dimensions shown in Figure 1, sequence is the one for which there are more experimentally determined data. This can be attributed to the increasing availability of sequencing methods, including recent advancements in metagenomic experiments, which enable the simultaneous sequencing of genomes from multiple species [55]. UniProtKB [1], a comprehensive database of protein sequences and associated biological knowledge, currently holds more than 246 million sequences belonging to more than 163 thousand proteomes (not considering redundant and excluded proteomes). At the same time, the MGnify database [56], which archives predicted protein sequences from the sequencing of metagenomic samples, contains more than 2.4 billion sequences.

The fraction of these proteins that has been experimentally characterised is minimal. Indeed, for most of the sequences in these databases, not even the existence of the protein has been confirmed, as protein sequences are solely predicted from genomic sequencing data. Swiss-Prot, a manually curated subset of UniProtKB that focus on well-studied organisms and proteins, contains 569 213 sequences (release 2023_1), or 0.23% of the total UniProt. Only 19.6% of the entries in Swiss-Prot contain evidence for the existence of the protein at the protein level, and another 9.8% at the transcription level. For TrEMBL, the non-curated portion of UniProt, these numbers are 0.08% and 0.55%, respectively. Finally, while the number of entries in Swiss-Prot has been essentially static for the past few years, TrEMBL and Mgnify have been growing exponentially, increasing the gap between the amount of raw sequence data and their functional characterisation.

Enzymes comprise 48.2% of Swiss-Prot (274 342), identified as all the entries associated with at least one EC number, while 15.5% (38.2 M) of TrEMBL entries are associated with an EC number. The enzyme coverage in Swiss-Prot and TrEMBL do not reflect the percentage of enzymes across life or even across the annotated species. On the one hand, there is an overrepresentation of enzymes in curated datasets such as Swiss-Prot, because enzymes have been more extensively studied in the past compared with other proteins. On the other hand, in non-curated datasets many proteins have not been assigned any function, including enzymes.

We have previously discussed the different ways enzymes are annotated in Swiss-Prot, and the data supporting these assignments, in the context of pseudoenzyme classification [57]. In that study, we verified that only 11% of Swiss-Prot entries contained experimental evidence related to the function of the protein, and of these, 46.9% (29 731) were labelled as enzymes. There is no better way to illustrate the challenge and the importance of computational methods to make correct functional assignments than contrast this number with the already mentioned number of non-redundant proteins in Mgnify, 2.4 billion, a difference of five orders of magnitude.

As discussed above, proteins (or domains) that evolve by duplication and divergence can be grouped in evolutionary families. Proteins in the same family can be identified by sequence similarity and typically perform the same or similar functions. These principles are the basis for several systems that try to organise the known protein sequence space. For example, the current version of Pfam [58] organises UniProt sequences into 19 632 related families of protein domains, based on matches to HMMs (hidden Markov models), representing each family. All sequences in a Pfam family share a common ancestor but they may not share the same function. Other notable classification systems of protein families include the CCD [59] and PantherDB [60]. PIRSF [61], rather than using domains, focus on the annotation of entire proteins. InterPro [62], which integrates these and other signatures in a single resource, assigns at least one signature to 84.1% of the sequences in UniProtKB. Evolutionary units smaller than the domain have also been identified and have been used to explain the evolutionary history and evolutionary relationships between domains [63].

### Structure

Protein structures are available through the Protein Data Bank (PDB) [2], a structural archive of biological macromolecules. As of June 2023, the PDB archives 206 462 structures, most of them containing proteins (201 976). In contrast with most protein sequences in UniProt, which are determined in bulk by genome sequencing of entire organisms, methods to solve protein structures (X-ray crystallography, Electron Microscopy and NMR) are costly and time intensive and so, are typically used to characterise one system at a time. For this reason, the size and growth of PDB is more modest than UniProt. PDB is also more redundant, since many structures deposited in the PDB belong to the same protein. The current 201 976 protein structures in PDB correspond to only 62 433 UniProt sequences. PDB is also biased to better studied organisms and proteins. Almost one third of the PDB structures are of human proteins, for example, and more than two thirds are enzymes.

#### Structural families and structure prediction

Structural similarity can also be used to find evolutionary relationships among proteins and to define families and, because protein structure is much more conserved than sequence, it allows us to retrieve much older relationships. It is possible to recognise homologous proteins based on their overall fold even when their sequences have diverged beyond recognition. SCOP [36], CATH [35] and ECOD [64] are well known structural classification systems that group evolutionary related proteins together. Taking CATH as an example, all protein domains within the same superfamily (such as CATH:3.40.50.720 — NAD(P)-binding Rossmann-like Domain) share a common ancestor, and have emerged by duplication and divergence, either by speciation events (leading to the appearance of orthologs) or gene duplication (leading to the appearance of paralogs). As of June 2023, CATH categorises more than 536 000 domains, belonging to more than 186 000 PDB structures, into 6631 distinct superfamilies.

For many years, template-based methods, which can predict a protein's structure starting from the structure of a homologous protein, were the most efficient tool to bridge the gap between the number of known sequences and the number of available structures [65]. Recently, deep-learning methods, most notably Alphafold [13], which only requires an alignment of homologous sequences to the query, have been able to generate high quality structural models, even for proteins that do not have a known structural homologue. The alphafold database [66] currently provides structural predictions for most sequences in UniProt. ESMFold, another deep-learning structure prediction method, has been used to predict the structure of more than 700 million metagenomic sequences [67].

### Ligand binding

The active site is the region of the enzyme where the reaction takes place. It needs to fulfil two main roles for the catalytic activity to happen: to bind the required substrates and co-factors; and to provide the catalytic residues and surrounding environment that are conducive to catalysis. When it comes to binding, there are two levels of knowledge that we might have for a given enzyme. The first is to know which ligands bind the enzyme and what is their binding affinity. Binding databases typically include data on both natural substrates and enzyme inhibitors. BrendaDB [68], a database containing kinetic information of enzyme reactions, contains 176 610, 69 886, and 46 076 values of *K*_M_, IC_50_, and KI, respectively. BindingMOAD [69] and PDBbind [70], which focus on complexes that exist in the PDB, contain affinity data for 15 223 and 19 443 complexes, respectively. BindingDB [71] has more than 2.7 million data points extracted from both academic papers and patents.

The second type of knowledge about binding is related to where the ligand binds in the active site and what are the conformations the ligand and the enzyme adopt upon binding. These types of data are ultimately derived from the PDB, since many enzymes in the database include ligands in their active site, but other databases curate this information in different ways. These include the sc-PDB [72], BioLip [73], the already mentioned BindingMoad and the NLDB [74], which also includes predicted complexes.

We have previously analysed how well the PDB covers the binding of native ligands to enzyme structures against the known reactions in EC and KEGG [75]. We found that most enzymatic structures in the PDB have either no ligand in the active site or a ligand with low similarity to the native one. Only 26% of the enzyme structures in the PDB bind a molecule that is at least 70% similar to the cognate ligand. This coverage increases to 58.9% and 62.9% if we aggregate all the structures belonging to the same KEGG reaction, or EC number, respectively. Nonetheless, this still means that there is no adequate enzyme–ligand structure for more than one third of the reactions curated in these databases.

#### Protein–ligand prediction

While Alphafold and similar methods helped filled the gap in structural coverage, when compared with sequence, and fixed some of the experimental biases in PDB, it did not help with the lack of enzyme–ligand structures, since its predictions do not include ligands. Alphafill [76] alleviates this problem somewhat by finding ligands that bind structurally similar protein regions in PDB and transposing these ligands to the Alphafold structure, but this solution does not extend to ligands that do not exist in the PDB.

When it comes to predict binding to uncharacterised proteins, there are at least three subproblems to solve, the identification of ligand binding sites, the identification of the correct ligands and their binding pose, and the estimation of the binding affinity. There are numerous computer tools dedicated to answer one or more of these questions [77,78]. Template-based methods work by looking at similar sequences or structures that have been previously characterised. Knowledge about the phylogenetic relationships can also be useful here, since it is expected that orthologous enzymes will bind the same substrates while paralogues might differ. Machine learning methods are also trained on existing data and can use both sequence or structural features to identify binding sites and potential ligands. Simulation methods, most notably molecular docking [79], can be used to predict binding poses and affinities *ab initio,* starting from the protein structure.

Despite the abundance of tools to predict protein–ligand binding, this is still an open problem. An accurate and general solution to identify good ligands for a given protein would be useful not only for the study of evolution and enzyme function but would be revolutionary for drug discovery, so progress in this area is bound to continue.

### Catalytic residues and co-factors

The catalytic residues are the amino acids in the active site of the enzyme that are responsible for accelerating the chemical reaction by lowering the energy of transition states or providing mechanistic paths that are not available elsewhere. The M-CSA (Mechanism and Catalytic Site Atlas) is the most comprehensive dataset of catalytic residues and includes curated annotations of the specific functions that the residues perform in each catalytic step. This data has been used in the past to better understand enzyme function and evolution. We recently did an overview of the frequency, roles and conservation of the catalytic residues across 648 enzyme families [3] and have also studied how mutations in the catalytic residues correlates with the evolution of pseudoenzymes [51,57]. The same dataset has also been used by others to answer biological questions [80,81], and to develop other data resources and methods [82–86], including most of the prediction methods discussed below.

Catalytic residues are extremely well conserved in evolution, even more so, in some examples, than the overall protein fold. For this reason, they are extremely important in the study of divergent evolution. Unlike the rest of the sequence, changes in catalytic residues are almost always associated with either a change or loss of catalytic function. Conversely, and unlike the overall protein sequence and structure, catalytic residues are also crucial to understand convergent evolution since the same active site composition and disposition can be found in unrelated enzymes.

We have recently reviewed the literature on studies and applications of 3D templates of catalytic residues [87], have analysed their flexibility in PDB structures [88], and their distribution in related and unrelated enzymes [89] using the M-CSA dataset. In related enzymes of both similar or divergent functions, active sites exhibit different degrees of structural variation, with the relative 3D disposition of catalytic residues being affected by their role in the mechanism and by binding of different substrates or products. With this geometric information we have generated several consensus templates representing compact clusters of catalytic residues. Recurring instances of these templates, which we have defined as the ‘3D modules of enzyme catalysis' [89], are typically associated with one or more functions and types of ligands and can themselves be used to better understand biological catalysis and evolution, and aid in enzyme design.

Co-factors are non-protein molecules that are required by many enzymes to perform their catalytic function. These molecules provide catalytic roles that cannot be performed by the canonical amino acids [90]. The evolutionary history of co-factors is interesting in its own right, since they are thought to be molecular fossils, catalysing reactions that can be traced back all the way to the origins of life. This argument has been initially made for nucleotide-like co-factors, which might be remnants of an RNA world [91], but has been extended to other organic and inorganic co-factors, which might have been the original catalysts in prebiotic geochemistry systems and later co-opted by RNA and protein-based enzymes [92]. Information about the roles of co-factors in enzyme mechanisms can be found in the M-CSA and the Co-factor database [4].

#### Prediction of catalytic residues

The identity of the catalytic residues of uncharacterised enzymes can be computationally inferred using both sequence and structural data. Due to their high conservation, a simple homology search and multi sequence alignment might be enough to identify potential catalytic residues, in particular, if the enzyme exists in distantly related species. In automated methods, conservation data is typically combined with other sequence-based features [93] and phylogenetic information [94,95]. Structurally, the clustering of catalytic residues in a well-defined pocket or cleft (the active site) and other features such as solvent accessibility, calculated pK_a_, and number of contacts, have been used by other methods [96,97]. It is also possible to create a network representation of the protein structure, which yields other descriptors such as closeness centrality that can also be used to distinguish catalytic residues [98]. Finally, some methods take an integrative approach by combining different types of data, typically with the help of machine learning algorithms to identify the best combination of features [99–103].

### Enzyme mechanism

The enzyme mechanism comprises all the atomic movements and bond changes in the active site that are responsible for moving the catalytic reaction forward. It is a crucial piece of data to understand how enzymes work and how they have evolved. The M-CSA (Mechanism and Catalytic Site Atlas) database [7] contains detailed annotations of the individual catalytic steps of 734 enzymes mechanisms. This is a small number when compared with the other databases mentioned in this review, which reflects both the lower number of studies in the literature and the complex nature of the problem, which requires many different types of data coming from different types of experiments. Nevertheless, since many related enzymes share the same mechanism, the coverage across the protein space is more extensive than it initially appears. By assuming that homologous sequences with the same set of catalytic residues and catalysing the same reaction also share the same mechanisms, the annotations in M-CSA can be extended to more than 15 000 PDB structures and 70 000 Swiss-Prot sequences.

The literature is lacking in studies looking at the evolution of enzymes at the mechanistic level. Twenty years ago, Bartlett et al. [40] performed a manual analysis of 27 pairs of homologous enzymes with different functions, to learn how they differed in catalytic residues and mechanisms. The picture for this small subset of enzymes was diverse. While all enzyme pairs had at least some active site similarities, only 15 pairs exhibited mechanism similarity. In this last group, enzymes shared some catalytic steps and diverged at others, and these changes, typically at the start or end of the mechanism, were enough to completely change the overall catalysed reaction. Another study [104] charted the appearance of new catalytic steps over evolutionary time to find that half of the observed chemistry in enzymes was already present in LUCA, while the other half appeared progressively over time.

**Figure 5.**
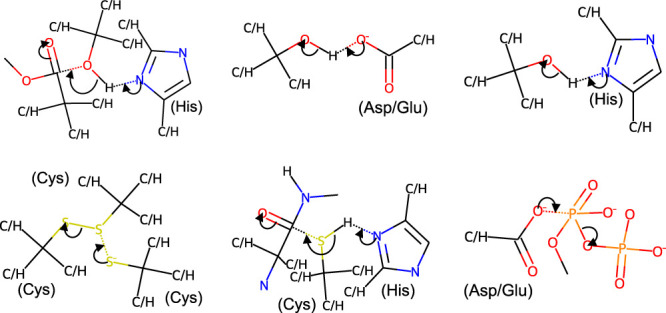
Some rules of enzymatic catalysis. Each rule represents a type of chemical step observed in one or more reaction mechanisms annotated in the M-CSA (Mechanism and Catalytic Site Atlas). We have previously described the process of creating these rules and their possible usages for studying enzyme evolution [105,134].

A large scale and complex analysis of the evolution of enzyme mechanisms has not been possible because until recently there was no way to automatically compare reaction mechanisms. We have recently generated a set of ‘catalytic rules' (see Figure 5) that are based on the catalytic steps annotated in M-CSA [105], which should be useful to automatically find similarities between the mechanisms of related and non-related enzymes.

#### Predicting the mechanism of enzymes

Simulation methods, such as QM/MM (Quantum Mechanism/Molecular Mechanics), are widely used to study the mechanism of enzymes *in silico* [106]. These methods provide a window to the active site by showing all catalytic events with atomic-level detail, something that is not accessible experimentally, due to the transient nature of the transition states and unstable intermediates. Although powerful, these methods are computationally expensive and difficult to setup, which limits their usage in large scale.

Homology can also be used to infer the mechanisms of enzymes but only if another enzyme with identical active site and function has already been characterised, in which case it can be assumed that both enzymes follow the same mechanism. To make use of the accumulated knowledge about enzyme mechanisms available in M-CSA, we have developed EzMechanism [105], a tool that can automatically generate mechanistic hypotheses for a given active site and chemical reaction. EzMechanism only takes into account local chemical similarities, so it also works for unrelated enzymes. Furthermore, it is able to compose mechanisms that have never been seen before. We are currently working on coupling the mechanistic hypotheses generated by EzMechanism to QM/MM calculations, with the objective of automatically describing their energetic profile.

### Enzyme reaction

Thousands of biological reactions have been identified, particularly those associated with the primary metabolism, and have been categorised by different resources. The EC list [37], the most widely used classification system for enzyme reactions, currently includes 6743 EC numbers [107]. KEGG Reaction [108] and Rhea [5], two databases of biological enzyme reactions, contain annotations for 11 858 and 15 116 distinct chemical reactions, respectively. EnviPath [109], which focuses on reactions involved in the biotransformation of environmental contaminants, contains 4398 reactions. Of the 569 793 sequences currently annotated in Swiss-Prot, 274 744 are annotated with an EC number [1]. In TrEMBL, which comprises the 248 M unreviewed sequences of UniProt, and where most of the annotation attributions have been done by homology, there are almost 40 M sequences associated with an EC number.

Like protein sequence and structure, chemical reactions can also be grouped by similarity, and the number of these clusters give a better indication of the size of the chemical space than the total number of reactions. For example, many enzymatic reactions describe the same transformation performed on molecules that share a common chemical group [39]. Typically, these arise from cases of divergent evolution, where binding residues are mutated, while catalytic residues, and the overall mechanism remain conserved. However, unlike sequences and structures, similar enzyme reactions can also be the result of convergent evolution, as discussed in the ‘Convergent Evolution' section.

A possible measure of the true diversity of enzyme reactions is the third level of the EC classification. As explained in the ‘Expansion of Enzyme Evolutionary Families' section, reactions that only differ on the fourth level can be considered the same type of reaction applied to a different substrate. According to this measure, the EC currently describes 316 types of reactions, or sub-subclasses. The KEGG database groups their reactions in 65 reaction classes defined by a transformation pattern, which can also be understood as types of reaction. Both the EC hierarchy and the definition of KEGG reaction classes, are manually curated. An automated analysis using EC-BLAST on a set of 6000 fully specified and balanced reactions built from the KEGG reaction database, was able to create clusters based on similar bond changes and reaction centres. In this analysis, more than 700 clusters with more than one reaction were created [110].

While the data discussed above focus on the classification of reactions and the identity of substrates and products, other databases such as Brenda [68] and Sabio-RK [111] hold information about the kinetics of reactions. Brenda contains more than 85 000 *k*_cat_ values while Sabio-RK contains more than 50 000 kinetic parameters, overall. Traditionally, using kinetic data for broad studies of enzyme function has been challenging because data has not been consistently provided in the literature. For example, in many papers of kinetic studies, the exact sequence of the protein being studied was either unknown, or not reported. Experimental conditions, which can greatly affect enzyme turn over, have also not been reported consistently. This problem has been addressed on recent years after the recognition of the importance of data standards. The STRENDA (Standards for Reporting Enzymology Data) guidelines, and their adoption by the main publications publishing enzymology studies, have been key to these advancements [112].

#### Prediction of enzyme reactions

Identifying the function of uncharacterised protein sequences and structures remains one of the most important outstanding goals in biology, and the number of existing tools to address this problem vast. As with the other dimensions, understanding conservation and neofunctionalization throughout evolution, is key to most of these prediction methods. Enzymes in the same family that contain the same conserved catalytic residues probably catalyse the same type of reaction, for example. The conservation of binding residues can further inform if the substrate specificities are the same. The existence of two orthologous enzymes in related species, and particularly when there are not paralogs, can also give a strong indication that both enzymes have the same function.

The CAFA (Critical Assessment of Functional Annotation) challenge is a competition aimed at evaluation existing computational tools for protein function prediction from sequence [113]. Methods are scored by how well they identify the most relevant GO terms for each sequence. In CAFA 3, the last challenge for which there is a report, the best methods at predicting the molecular function ontology were GoLabeler (now superseded by NetGo 3.0) [114] and CATH funfams. Both of these tools, as well as more recently developed methods, such as ProteinBert [115] and DeepGo [116], combine traditional sequence similarity methods with varied machine-learning approaches that are able to identify function-defining residues or motifs. Similar approaches do exist specific to enzymes, where the goal is to predict an EC number. A non-exhaustive list includes EzyPred [117], DEEPre [118], ECPred [119], and DeepEC [120]. Structural information is also considered by other methods, such as ProFunc [121], CO-FACTOR [122], and DeepFRI [123]. While using structure to predict function was traditionally less useful than using solely sequence, due to the limited availability of protein structures, this might now change with the ease of generating good structures for most sequences. Finally, information specific to the catalytic sites can also be used [124]. Methods that use templates of catalytic residues should be able to detect active site similarities in related but also unrelated enzymes when the catalytic residues converged to the same geometry [89].

By design, the methods discussed above are limited to identify chemical reactions that are already annotated in the classification system. Furthermore, most methods using sequence information together with machine learning lack interpretability, making the evaluation of assignments for specific enzymes tricky. Considering the methodologies described in the previous sections to predict the intermediate dimensions from sequence, it should be also possible, in principle, to predict the reactions of enzymes *ab initio*, in a way that is not limited to existing reactions. Alphafold and similar methods are already able to satisfactorily predict the structure of proteins from sequence. If predicting ligand binding and the enzyme mechanism becomes straightforward in the same manner, it will be possible to predict the reaction from sequence while establishing a clear causality chain across the six dimensions discussed here.

## Conclusion

Theodosius Dobzhansky famously stated that ‘Nothing in Biology Makes Sense Except in the Light of Evolution'. This is clearly the case for enzymes, for which catalytic sites can be found conserved between bacteria and humans, and possible catalytic reactions can only arise by evolutionary paths that navigate the complex protein space while being nudged by epistatic and pleiotropic effects.

Most new catalytic reactions arise as a result of gene duplication and divergence. Changes in substrate specificity can be traced to changes in the binding residues and typically correspond to a last-digit modification of the EC number. Changes in the catalytic residues are much rarer but can lead to completely new chemical activities. Studies to understand the precise evolutionary processes of neofunctionalization are still lacking, in particular the role of mutations in the catalytic residues and changes in the enzyme mechanism. Ideally, we would like to classify all functional changes across several enzyme families according to the paradigms shown in Figure 3 (together with other possibilities).

Neutral drift and enzyme promiscuity have an important role in exploring the catalytic space without impacting fitness. Promiscuous functions can become adaptive after an environment or cellular change, and while initially inefficient, their activity can be improved after duplication and specialisation. The extent at which promiscuity is relevant for enzyme function and evolution has been recognised for several examples, but these kinds of data have not yet been used for large-scale computational studies, since available information and in its curation in databases is still limited [125].

The evolution of new enzymes through recombination of domains is another area that, in our opinion, would benefit from further studies. As explained above, domain recombination allows for big jumps in function and is particularly relevant for co-factor-containing enzymes. Studies focusing on the contribution of each domain for binding, catalytic residues, and the catalytic mechanism, would be helpful to understand domain recruitment during evolution.

In this review, we highlighted some databases with information about enzymes as well as some computational methods that can be used to close gaps in knowledge. These examples were meant to illustrate some of the available tools but are by no means exhaustive. Similarly, in the interest of brevity, other topics pertinent to enzyme function and evolution such as metabolic databases, enzyme engineering, in particular directed evolution and rational design, ancestral reconstruction, and the role of structural dynamics, have been excluded from the discussion.

One of the challenges of studying enzymes from the point of view of bioinformatics is related with the variety of the available data, which mirrors the complexity of enzymes as biological catalysts The integration of all these kinds of data, which we have organised here across six dimensions, is necessary to explain enzyme function and evolution, and crucial for efforts of enzyme design. Another challenge is the limited availability of data for certain dimensions, particularly when compared with the number of protein sequences. Our optimistic viewpoint is that by using different computational approaches, including template-based, machine learning, and simulation methods, it will be possible in the future to have a comprehensive knowledge base of enzyme function and evolution across these dimensions. In our opinion, the biggest obstacles to this vision are currently the prediction of protein–ligand binding and of the enzyme reaction mechanism.

## Abbreviations

EC: enzyme commission
IAD: Innovation–Amplification–Divergence
LUCA: Last Universal Common Ancestor
PDB: Protein Data Bank
